# Multiscale spectroscopic analysis of lipids in dimorphic and oleaginous *Mucor circinelloides* accommodate sustainable targeted lipid production

**DOI:** 10.1186/s40694-023-00148-z

**Published:** 2023-01-16

**Authors:** V. Shapaval, A. Deniset-Besseau, D. Dubava, S. Dzurendova, J. Heitmann Solheim, A. Kohler

**Affiliations:** 1 Faculty of Science and Technology, Norwegian University of Life Sciences, P.O. Box 5003, 1432 Ås, Norway; 2 Laboratoire de Chimie Physique, CNRS, Université Paris-Saclay, 91405 Orsay, France

**Keywords:** Vibrational spectroscopy, Lipid droplets, Dimorphic fungi, Nanospectroscopy, Microspectroscopy

## Abstract

**Background:**

Oleaginous fungi have versatile metabolism and able to transform a wide range of substrates into lipids, accounting up to 20–70% of their total cell mass. Therefore, oleaginous fungi are considered as an alternative source of lipids. Oleaginous fungi can accumulate mainly acyl glycerides and free fatty acids which are localized in lipid droplets. Some of the oleaginous fungi possessing promising lipid productivity are dimorphic and can exhibit three cell forms, flat hyphae, swollen hyphae and yeast-like cells. To develop sustainable targeted fungal lipid production, deep understanding of lipogenesis and lipid droplet chemistry in these cell forms is needed at multiscale level. In this study, we explored the potential of infrared spectroscopy techniques for examining lipid droplet formation and accumulation in different cell forms of the dimorphic and oleaginous fungus *Mucor circinelloides*.

**Results:**

Both transmission- and reflectance-based spectroscopy techniques are shown to be well suited for studying bulk fungal biomass. Exploring single cells with infrared microspectroscopy reveals differences in chemical profiles and, consequently, lipogenesis process, for different cell forms. Yeast-like cells of *M. circinelloides* exhibited the highest absorbance intensities for lipid-associated peaks in comparison to hyphae-like cell forms. Lipid-to-protein ratio, which is commonly used in IR spectroscopy to estimate lipid yield was the lowest in flat hyphae. Swollen hyphae are mainly composed of lipids and characterized by more uniform distribution of lipid-to-protein concentration. Yeast-like cells seem to be comprised mostly of lipids having the largest lipid-to-protein ratio among all studied cell forms. With infrared nanospectroscopy, variations in the ratios between lipid fractions triglycerides and free fatty acids and clear evidence of heterogeneity within and between lipid droplets are illustrated for the first time.

**Conclusions:**

Vibrational spectroscopy techniques can provide comprehensive information on lipogenesis in dimorphic and oleaginous fungi at the levels of the bulk of cells, single cells and single lipid droplets. Unicellular spectra showed that various cell forms of *M. circinelloides* differs in the total lipid content and profile of the accumulated lipids, where yeast-like cells are the fatty ones and, therefore, could be considered as preferable cell form for producing lipid-rich biomass. Spectra of single lipid droplets showed an indication of possible droplet-to-droplet and within-droplet heterogeneity.

## Background

Oleaginous fungi are able to accumulate lipids at 20–70% of their total cell mass [1]. Fungal biomass is an emerging alternative source of lipids. Oleaginous fungi accumulate lipids in the form of triacylglycerides (TAGs) with fatty acid profiles similar to the plant and/or fish oils, and that are localized in globular intracellular organelles called lipid droplets or lipid bodies [2, 3]. Many oleaginous fungi are dimorphic microorganisms and may exhibit different cell morphologies: unicellular yeast-like cells and multicellular pseudo-hyphae or hyphae [4]. Industrially important oleaginous fungi such as *Yarrowia lipolytica*, *Trichosporon cutaneum* and *Mucor circinelloides* exhibit morphological switch depending on the environmental conditions. The molecular rationale for this dimorphic switch in oleaginous fungi is little explored, and the mechanisms of lipid storage, content and lipid profiles in different cell forms are yet to be elucidated. Thus, to provide a deeper understanding of lipid storage and lipid droplet formation mechanisms in fungal cells of different morphologies, there is a need for applying a multiscale analytical approach to evaluate fungal biomass on the scale of bulk cells (macroscale), as well as to study lipid formation at cellular (microscale) and subcellular (nanoscale) level. In addition, information on the total lipid content of the bulk fungal biomass at macroscale level needs to be further connected with the lipid droplet distribution and composition in single fungal cell forms at microscale level, and organization of lipids within the single lipid droplets at nanoscale level.

Traditional macroscale lipid analysis of bulk cells is generally performed using solvent-based lipid extraction with the subsequent determination of fatty acid profiles by gas chromatography (GC) techniques. This approach is time-consuming and therefore not suitable for high-throughput screening or monitoring of lipid production processes in dimorphic fungal cells. Since traditional lipid analysis involves a multi-step preparation procedure and extraction, operation errors can occur resulting in reduced reproducibility of the method [5–7]. Micro- and nanoscale analysis of lipids can be done by the advanced microscopy techniques such as transmission electron microscopy (TEM) [8, 9], freeze-fracture and low temperature scanning electron microscopy (SEM) [10], and non-linear optical (NLO) microscopy techniques such as third harmonic generation (THG) microscopy [11, 12]. However, none of those techniques provide information about the chemical composition of lipid droplets at the micro- and nanoscale level in a non-destructive way.

Infrared spectroscopy is a biophysical technique that offers a unique opportunity for performing non-destructive multiscale lipid analysis. Today, it is a state-of-the-art technique that has been widely used for the classification, identification and characterization of bacteria [13, 14], yeast [3, 15–19], and filamentous fungi [1, 20–26]. Numerous studies have been conducted in recent years to investigate fungal lipid biology using macroscale Fourier transform infrared (FTIR) spectroscopy techniques [22–26]. High correlation between infrared (IR) and lipid GC data allowed building calibration models for the prediction of total lipid content and fatty acid profiles in oleaginous fungi, and identification of different classes of fatty acids [22, 23]. Furthermore, IR spectroscopy has proven itself as a highly reproducible analytical tool for high-throughput investigation of the effect that temperature, carbon to nitrogen ratio (C/N ratio), nitrogen and phosphorus sources have on lipid production in oleaginous filamentous fungi [6, 24].

The characterization of total lipid content, lipid profiles, and the distribution of lipid bodies at the single cell level can be obtained using FTIR microspectroscopy, which has gained widespread acclaim as an analytical tool for the microscale single cell analysis. Over the past decades, FTIR microspectroscopy measurements of single cells have been employed for the chemical analysis of algae [27] and to some extent filamentous fungi [28, 29], while it has not been applied so far for characterizing dimorphic oleaginous fungi. To explore the subcellular organization of fungal hyphae and specify lipid composition at the level of single lipid droplets, atomic force microscope infrared spectroscopy (AFM-IR), a technique that combines the descriptive strength of infrared spectroscopy with the accuracy of an atomic force microscope [30], can be used. AFM-IR enables chemical profiling of the cell wall, cell membrane, lipid droplets and other organelles at the nanoscale level [30], and, thus, provides a unique opportunity for obtaining new information related to the subcellular organization in the dimorphic fungi.

The aim of this study was to evaluate the applicability of IR techniques for lipid analysis at different scales in oleaginous dimorphic fungi and, further, for the first time, to investigate data compatibility between different measurement scales. We have chosen a dimorphic oleaginous *Mucor circinelloides* as a model oleaginous microorganism, which may be present in the form of single yeast-like cells, flat and swollen hyphae. For this study, we have employed FTIR spectroscopy measurements at different scales of structural organization: bulk cells (high-throughput screening (HTS)- and attenuated total reflectance (ATR)-FTIR spectroscopy), single yeast-like and hyphae cells (FTIR microspectroscopy), and single lipid droplets (AFM-IR nanospectroscopy). Since lipids may be subjected to storage-related changes such as oxidation in the case of unsaturated TAGs, it is important to have knowledge about the chemical stability of the fungal biomass. Therefore, chemical stability of fungal biomass was also examined by means of ATR-FTIR spectroscopy to evaluate the influence of long-term storage on lipid content in fungal cells. To the authors' knowledge, this is the first research to employ FTIR microspectroscopy and AFM-IR nanospectroscopy to study the distribution and composition of lipid droplets in filamentous fungi that are both oleaginous and dimorphic.

## Results

### Analysis of lipid content by HTS- and ATR-FTIR spectroscopy of bulk cells

**Fig. 1:**
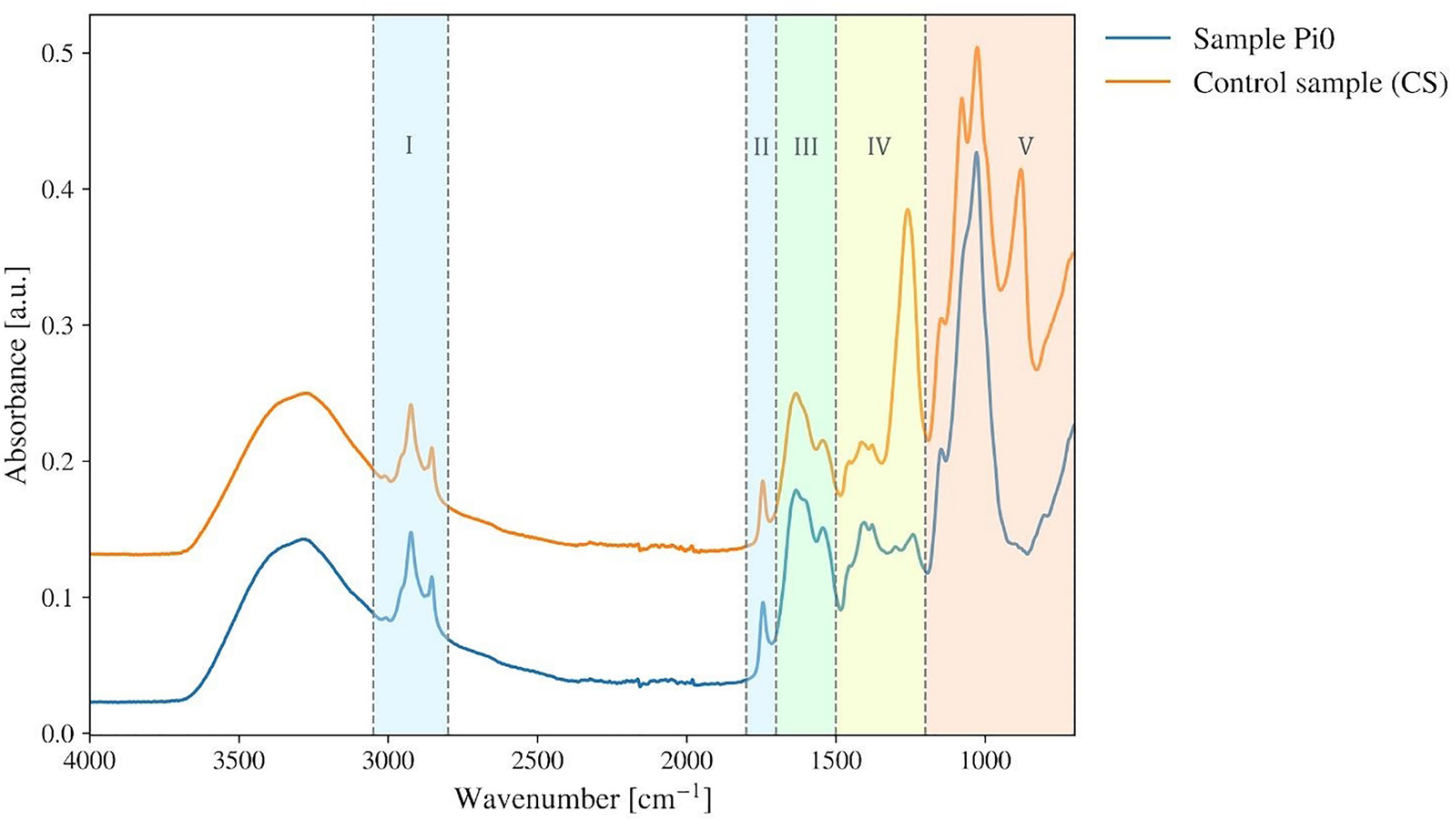
EMSC-preprocessed ATR-FTIR spectra of *Mucor circinelloides* fungus grown under different cultivation conditions: absence of inorganic phosphorus (sample Pi0) and presence of inorganic phosphorus (sample Pi1 or control sample—CS). The following spectral regions are colored: I and II—lipids (3050–2800 cm^−1^ and 1800–1700 cm^−1^ respectively), III—proteins (1700–1500 cm^−1^), IV—mixed region (1500–1200 cm^−1^), V—polysaccharides + polyphosphates (1200–700 cm^−1^)

For the macroscale evaluation of total lipid content in cell populations of *Mucor circinelloides*, two IR spectroscopy techniques, namely HTS-FTIR and ATR-FTIR spectroscopy, were compared. HTS-FTIR spectroscopy has been widely utilized by researchers for the non-destructive analysis of lipids in populations of oleaginous microorganisms, and a measurement protocol is readily available, and it was validated in the previous studies [22–24]. At the same time, the use of ATR-FTIR spectroscopy for the study of cellular lipids in microorganisms is reported less frequently [31]. While ATR-FTIR is not a high-throughput technique, it has some advantages related to spectral signal standardization. We employed ATR-FTIR spectroscopy to check that dried fungal biomass used for the infrared measurements is chemically stable over a time typically needed for low-throughput infrared experiments. Therefore, a simple protocol for the analysis of lipids in oleaginous fungi by ATR-FTIR spectroscopy was developed, and the hypothesis that sample lipid composition does not significantly change with the storage time upon drying was tested. According to the protocol, ATR-FTIR spectra were acquired from the non-disintegrated fungal biomass that had been washed with distilled water and dried at room temperature (Fig. 1). Storage time prior to ATR-FTIR measurements varied from one to 7 days. We investigated the influence of storage time on ATR-FTIR spectral biochemical profiles of *M. circinelloides* biomass, which was obtained after the cultivation in the media with limited nitrogen and different amounts of inorganic phosphorus (Pi) used for triggering lipid accumulation (Table 1) as described by Dzurendová et al. [6, 24]. Spectral fingerprints of fungi grown under different conditions are distinguishable from each other and can therefore be used as a reference to estimate and compare the variance introduced to infrared spectral data by the storage time. The effect of both factors, i.e. storage time and growth conditions, on the infrared spectral profiles of fungal biomass was estimated by ANOVA-PCA.

**Table 1:** Concentration of phosphate salts added to the medium used for the cultivation of *Mucor circinelloides* fungi for HTS/ATR-FTIR spectroscopy, FPA-FTIR microspectroscopy and AFM-IR nanospectroscopy

Sample name	KH_2_PO_4_ (g L^−1^)	Na_2_HPO_4_ (g L^−1^)
Pi4	28	8
PI2	14	4
Pi1	7	2
Pi0.5	3.5	1
Pi0.25	1.75	0.5
Pi0	0	0

**Fig. 2:**
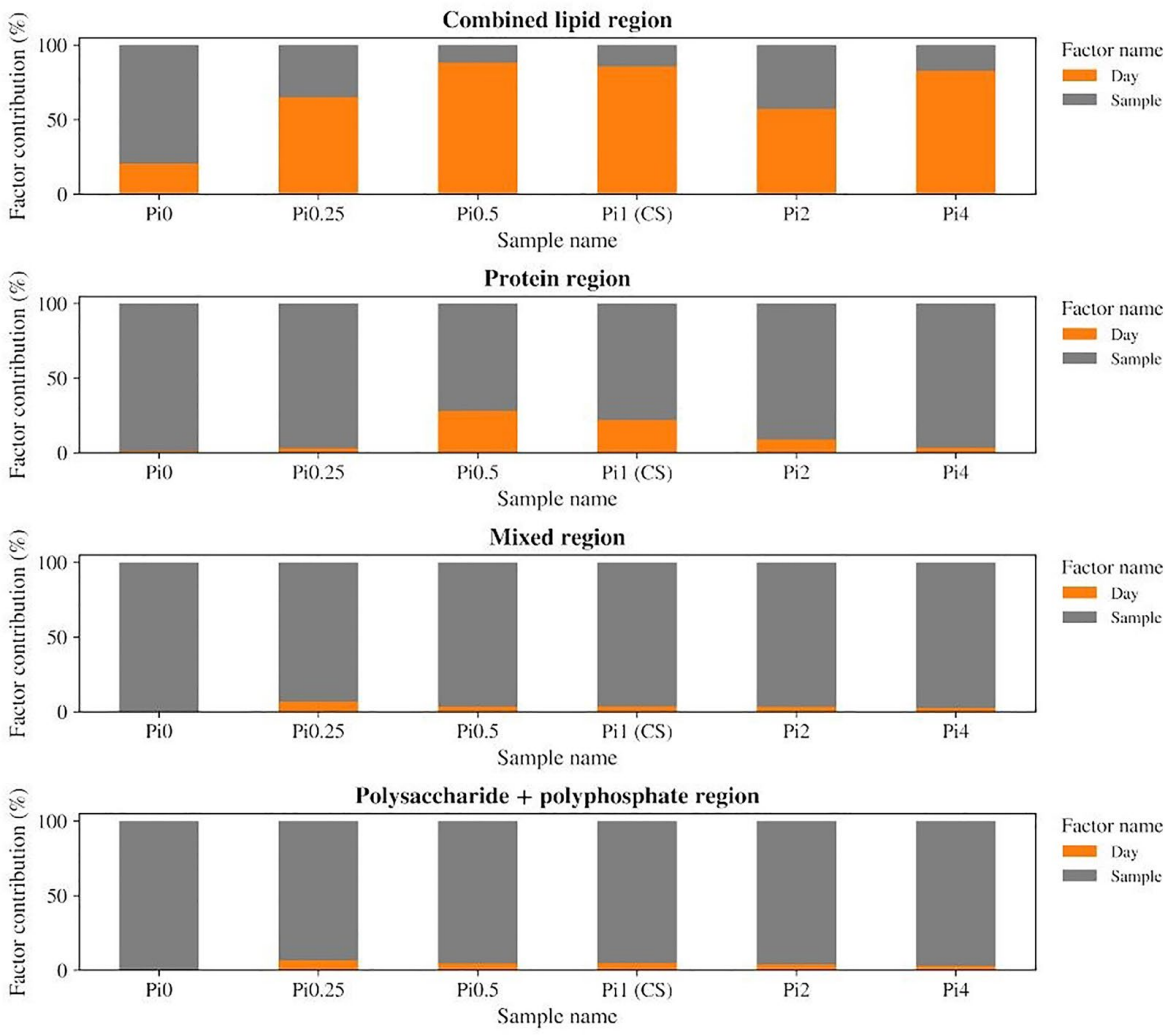
Contribution of storage time and cultivation condition factors in ANOVA model of ATR-FTIR spectra of *Mucor circinelloides* fungus, spectral regions: (1) Combined lipid region: 3050–2800 cm^−1^ and 1800–1700 cm^−1^, (2) Protein region 1700–1500 cm^−1^, (3) Mixed region: 1500–1200 cm^−1^, (4) Polysaccharide + polyphosphate region: 1200–700 cm^−1^. Each bar corresponds to a particular sample

**Fig. 3:**
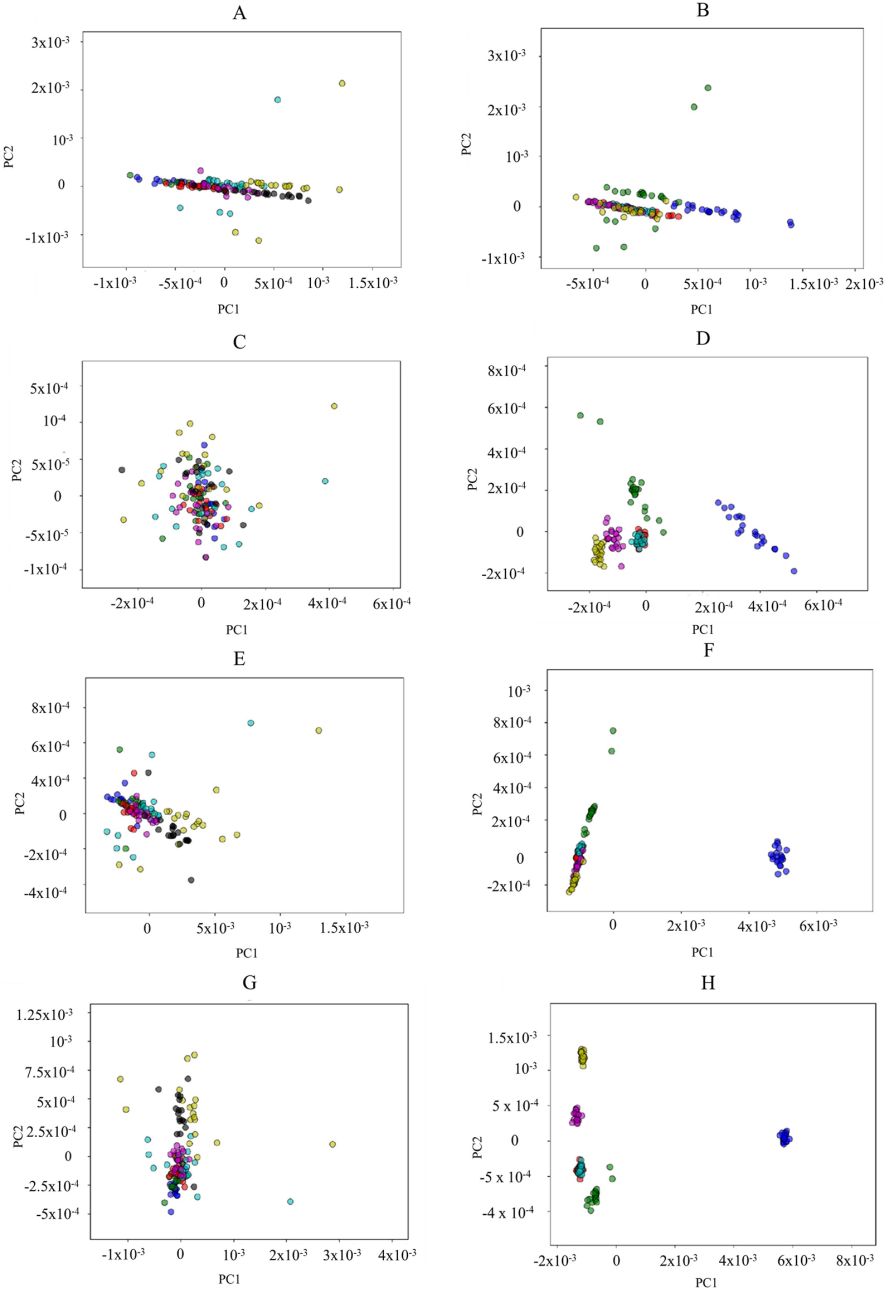
ANNOVA-PCA score plots of ATR-FTIR spectra of *Mucor circinelloides* fungus, spectral regions such as lipid regions (**A**) and (**B**)—3050–2800 cm^−1^ and 1800–1700 cm^−1^, protein (**C**) and (**D**)—1700–1500 cm^−1^, mixed (**E**) and (**F**)—1500–1200 cm^−1^, polysaccharide + polyphosphate region (**G**) and (**H**) 1200–700 cm^−1^. Score plots (**A**, **C**, **G**) showing different days where "blue"—Day 1, "green"—Day 2, "red"—Day 3, "light blue"—Day 4, "violet"—Day 5, "yellow"—Day 6, "black"—Day 7 and (**B**, **D**, **F**) and (**H**) showing different samples where "blue"—Sample Pi0, "green"—Sample Pi0.5, "red"—Sample Pi0.5, "light blue"—Sample Pi1, "violet"—Sample Pi2, "yellow"—Sample Pi5

ANOVA-PCA of four different spectral regions, namely 3050–2800 cm^−1^ and 1800–1700 cm^−1^ (combined lipid region), 1700–1500 cm^−1^ (protein region), 1500–1200 cm^−1^ (mixed region), 1200–700 cm^−1^ (polysaccharide and polyphosphate region) was performed. It has been shown that storage time does not have a long-term effect on the chemical composition of *M. circinelloides* biomass with respect to its protein, polysaccharide, and polyphosphate content (Fig. 2). At the same time, it can be seen, that ANOVA-PCA score plots of the lipid region (Fig. 3A, B) did not show any differences that could be utilized for differentiation according to both sample and storage time. It should be noted that ATR-FTIR spectra of biomass after 6 and 7 days of drying were found to be visually more separated from the spectra of other samples on ANOVA-PCA score plots corresponding to lipid, mixed, and polysaccharide combined with polyphosphate spectral regions (Fig. 3A, E, G). It can be speculated that fungal biomass might undergo minor time-dependent changes in its lipid and polysaccharide composition associated with the amount of water in the sample that became more pronounced after prolonged storage. As it has been suspected, no differences caused by varying storage time could be seen on ANOVA-PCA score plots corresponding to the protein spectral region (Fig. 3C), due to protein content being the most stable in the cells. In addition to that, the chemical profile of sample Pi0 differs from other samples due to it was obtained from the cultivation in nitrogen-limited media but without addition of inorganic phosphorus, that could lead to the distinct changes in the cells' chemistry (Fig. 3D, F, H). It should be noted that the best separation of different samples according to the cultivation conditions was achieved using mixed spectral region (Fig. 3G, H), that could be due to a large contribution of a polyphosphate peak at 1265 cm^−1^. Since all samples have been grown under different phosphorus concentrations in media influencing the amount of polyphosphates stored in the fungal cells, variation in the absorbance of phosphate-related peaks might be what distinguishes them from each other.

**Fig. 4:**
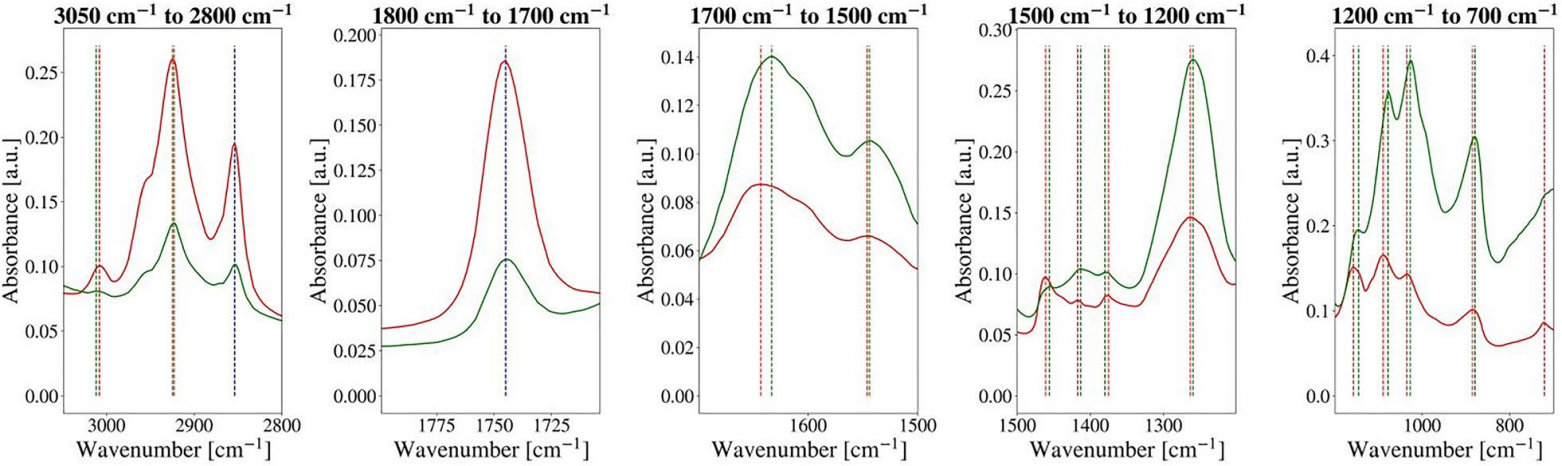
HTS-FTIR (red) and ATR-FTIR (green) spectra of *Mucor circinelloides* fungi, sample Pi1 (CS). The scale of HTS-FTIR spectra was adjusted to match the scale of ATR-FTIR spectra Red dotted lines represent absorbance peaks in HTS-FTIR spectra, green—in ATR-FTIR spectra and blue—matching peaks in both

Compatibility between lipid-related information obtained from HTS- and ATR-FTIR spectra of *M. circinelloides* was evaluated by analyzing the following lipid-associated absorption peaks (Table 2): =CH stretching at 3010 (± 6) cm^−1^, CH_2_ and CH_3_ stretching at 2925 (± 6) cm^−1^ and 2855 (± 6) cm^−1^, ester bond vibrations at 1742 (± 6) cm^−1^, CH_2_ and CH_3_ bending at 1465 (± 6) cm^−1^ and 1375 (± 6) cm^−1^, and C–H deformation vibration at 1415 (± 6) cm^−1^ (Table 2, Fig. 4). The selection of peaks was based on HTS-FTIR spectra as it is the most common measurement mode for macroscale FTIR analysis of fungal cells. Numbers in brackets are given to emphasize uncertainty in the measured peak positions due to the resolution of a spectrometer allowing to separate two adjacent spectral lines.

**Table 2:** Main peaks in HTS-FTIR spectra of fungi

Spectral range	Wavenumbers (cm^−1^)	Group and class	Assignment	Remarks
Lipid region I (3050–2800 cm^−1^)	2955	CH_3_ functional group in aliphatic compounds	C-H asymmetrical stretching	Hydrocarbon chain length in lipids
	2930, 2925	> CH_2_ functional group in aliphatic compounds		
	2885–2865 (2855 in our case)	CH_3_ functional group in aliphatic compounds	C-H symmetrical stretching	Hydrocarbon chain length in lipids
	2870–2840	> CH_2_ functional group in aliphatic compounds		
	3010	=C-H functional group in aliphatic compounds	=C–H stretching	Unsaturation index in lipids
Lipid region II (1800–1700 cm^−1^)	1745, 1742	Carboxylic group in triglycerides	C=O stretching	Total lipid content (frequency decreases with chain length)
	1708, 1710	C=O group in carboxylic acid dimers	C=O stretching	Free fatty acids
Amide region (1700–1500 cm^−1^)	1670–1630 (1650)	C=O groups in primary and secondary amides	C=O stretching	Amide I band
	1610–1570	Carboxylate group	Antisymmetric COO^−^ stretching	Ionized free fatty acids
	1560–1530	–CO–NH– groups in primary and secondary amides	Coupled in-plane N–H bending and C–N stretching	Amide II band
Mixed region (1500–1200 cm^−1^)	1480–1440	Methyl groups	CH_3_ antisymmetric bending	Lipids
	1480–1440	Methylene groups	CH_2_ bending	Lipids
	1380–1360	Aliphatic compounds	Symmetric CH_3_ bending	Lipids
	1280–1070	C–O–C in esters, ethers, lactones	C–O–C antisymmetric stretching	Polysaccharides
Carbohydrate region (1200–700 cm^−1^)	1280–1220	Phosphodiester bonds in nucleic acids	P=O asymmetrical stretching	Polyphosphate
	1050–1000	Alkyl aryl ethers	C–O–C stretching	Polysaccharides
	740–720	Methylene groups in aliphatic compounds (hydrocarbons and lipids)	CH_2_ rocking in hydrocarbon chains	Intensity increases with the increasing chain length

**Fig. 5:**
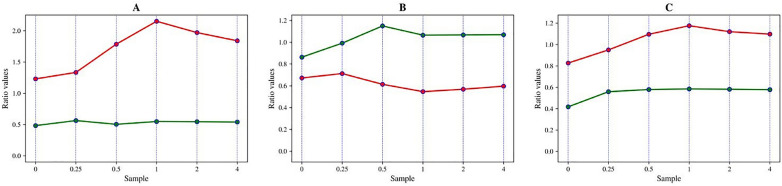
Peak ratios of HTS-FTIR (red) and ATR-FTIR (green) spectra of *Mucor circinelloides* fungi: **A** 1742 cm^−1^ vs 1650 cm^−1^, **B** 3010 cm^−1^ vs. 1742 cm^−1^, **C** 3010 cm^−1^ vs. 1650 cm^−1^

By overlapping pre-processed HTS- and ATR-FTIR spectra, it has been shown that all lipid-associated peaks present in HTS-FTIR spectra were present in ATR-FTIR spectra as well (Fig. 4). However, differences in the peak positions between HTS- and ATR-FTIR peaks corresponding to the same molecular group vibrations could be seen throughout the whole spectral range (Fig. 5). Peaks in the low frequency range of the mid-infrared range (400 cm^−1^) appeared to exhibit larger shifts compared to peaks in the high frequency range (3200 cm^−1^). The most pronounced shifts were observed for the peaks at 1465 (± 6) cm^−1^, 1415 (± 6) cm^−1^ and 1375 (± 6) cm^−1^, while other lipid-related peaks (3010 (± 6) cm^−1^, 2925 (± 6) cm^−1^, 2855 (± 6) cm^−1^, and 1742 (± 6) cm^−1^) showed little to no shift. Furthermore, HTS-FTIR spectra were characterized by higher absorption intensities for the selected lipid peaks closer to the higher wavenumber side of the spectrum in comparison to ATR-FTIR spectra. For example, the peaks at 3010 (± 6) cm^−1^, 2925 (± 6) cm^−1^, 2855 (± 6) cm^−1^, and 1742 (± 6) cm^−1^ were shifted to higher frequencies in HTS-FTIR spectra compared to ATR-FTIR spectra, while HTS-FTIR absorption values for peaks at 1415 (± 6) cm^−1^ and 1375 (± 6) cm^−1^ appeared lower than corresponding ATR-FTIR absorption values (Fig. 4). As a consequence, ratios between different lipid-related peaks (i.e. 3010 cm^−1^ vs. 1742 cm^−1^) in HTS- and ATR-FTIR spectra differed from one another as well (Fig. 5), where in most of the cases, HTS-FTIR absorbance ratio values were higher than ATR-FTIR ratio values.

### Microscale profiling of accumulated lipids in different cell forms of dimorphic *Mucor circinelloides* by FTIR microspectroscopy

The use of an FTIR spectrometer coupled to a microscope with infrared optics allows performing chemical analysis of lipids stored in fungi at the single cell level and examining diversity in lipid accumulation for different cell morphologies.

**Fig. 6:**
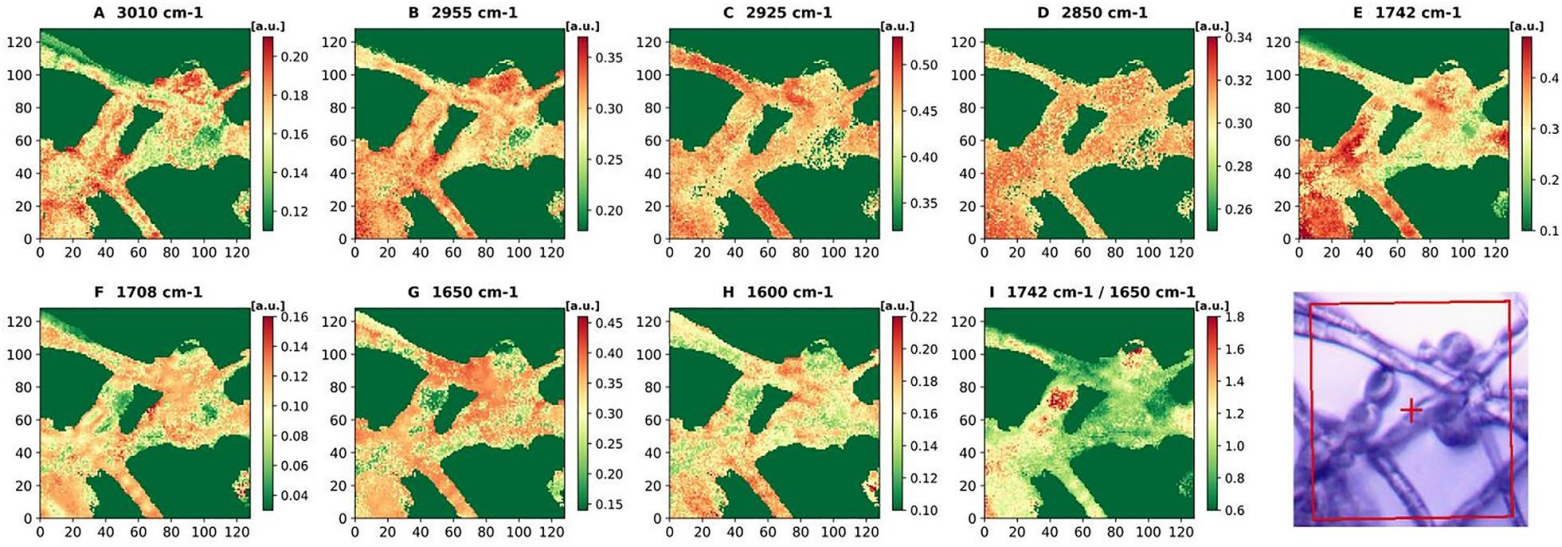
FPA-FTIR spectral maps (**A**–**I**) and optical image of flat hyphae of *Mucor circinelloides* fungus, sample Pi1 (CS): **A** 3010 cm^−1^, **B** 2955 cm^−1^, **C** 2925 cm^−1^, **D** 2850 cm^−1^, **E** 1742 cm^−1^, **F** 1708 cm^−1^, **G** 1650 cm^−1^, **H** 1600 cm^−1^, **I** 1742 cm^−1^ vs 1650 cm^−1^

**Fig. 7:**
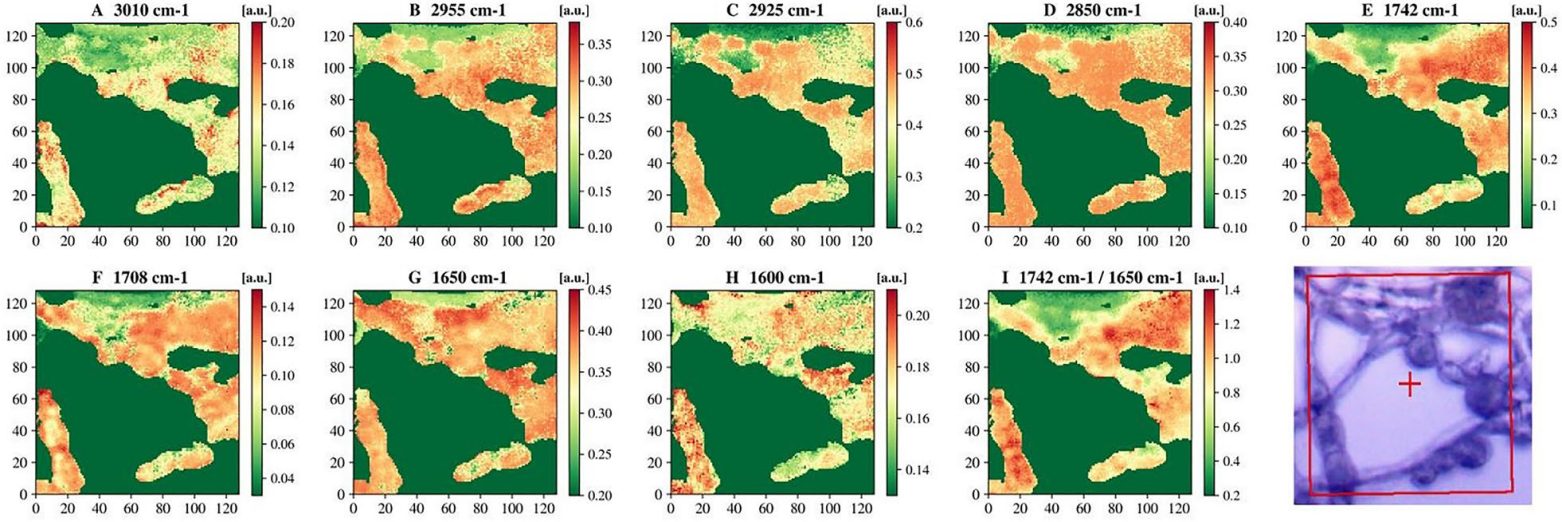
FPA-FTIR spectral maps (**A**–**H**) and FPA optical image of swollen hyphae of *Mucor circinelloides* fungus sample Pi1 (CS): **A** 3010 cm^−1^, **B** 2955 cm^−1^, **C** 2925 cm^−1^, **D** 2850 cm^−1^, **E** 1742 cm^−1^, **F** 1710 cm^−1^, **G** 1650 cm^−1^, **H** 1600 cm^−1^, **I** 1742 cm^−1^ vs. 1650 cm^−1^

**Fig. 8:**
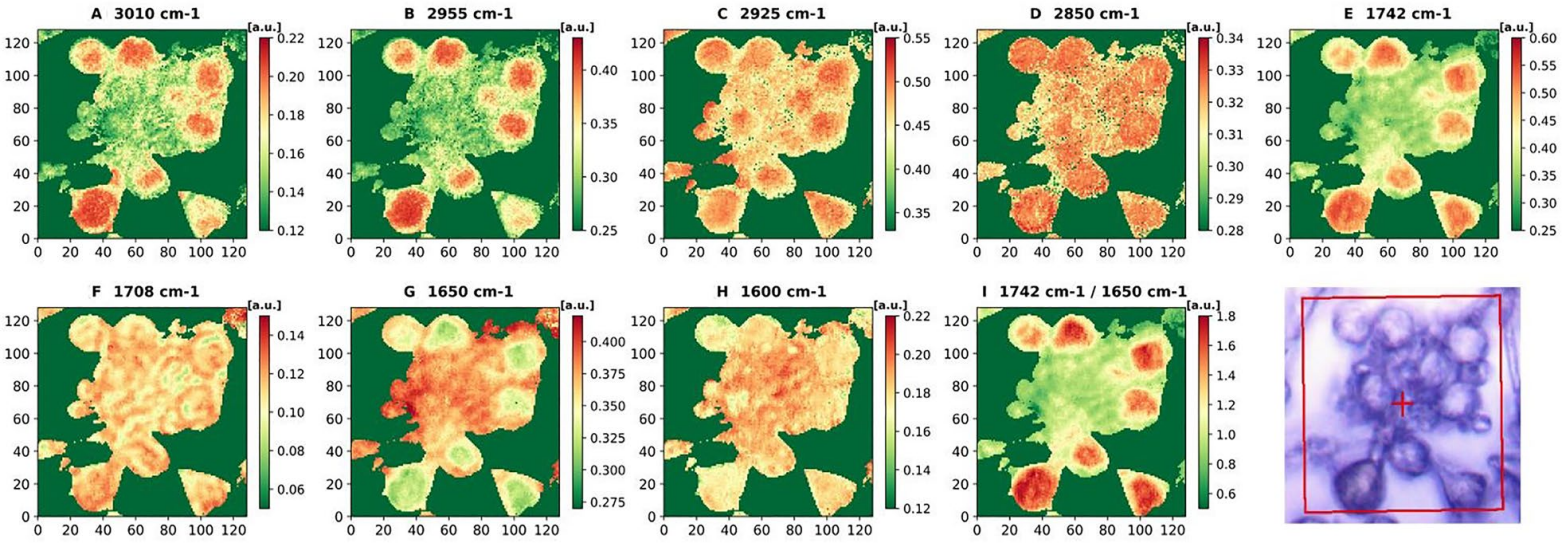
FPA-FTIR spectral maps after EMSC correction (**A**–**H**) and FPA optical image of yeast-like cells of *Mucor circinelloides* fungus, sample Pi1 (CS): **A** 3010 cm^−1^, **B** 2955 cm^−1^, **C** 2925 cm^−1^, **D** 2850 cm^−1^, **E** 1742 cm^−1^, **F** 1710 cm^−1^, **G** 1650 cm^−1^, **H** 1600 cm^−1^, **I** 1742 cm^−1^ vs. 1650 cm^−1^

A control sample of *M. circinelloides* (Pi1) which showed to be mixture of all three cell forms—yeast-like cells, flat and swollen hyphae was selected to perform microscale analysis of lipid accumulation in different cell forms by means of FPA-FTIR microspectroscopy. At the stationary growth phase under conditions triggering lipid accumulation (nitrogen limitation), *M. circinelloides* has been shown to exhibit dimorphism in three types of cell forms: hyphae-like cells (flat hyphae and swollen hyphae—Figs. 6 and 7) and yeast-like cells (Fig. 8), which appeared in the same culture. We studied FPA-FTIR spectral maps for the main lipid-associated peaks, namely 3010 (± 4) cm^−1^, 2955 (± 4) cm^−1^, 2925 (± 4) cm^−1^, 2850 (± 4) cm^−1^, 1742 (± 4) cm^−1^, 1708 (± 4) cm^−1^, as well as a protein-associated peak at 1650 (± 4) cm^−1^, and calculated peak ratio maps (TAG-associated peak at 1742 cm^−1^ vs. protein-associated peak at 1650 cm^−1^) for all three types of cell forms. A spectral map for 1600 cm^−1^, which could be seen as a shoulder in both HTS- and ATR-FTIR spectra, was investigated as well. It was not possible to find a relatively large area that would account for one of the cell forms only, therefore each selected map represents the predominant cell form. Despite that, different cell forms of *M. circinelloides* showed significant differences in the absorbance intensities for lipid-associated peaks (Figs. 6, 7 and 8).

The flat and swollen hyphae showed lower absorbance intensities for all lipid-associated peaks in comparison to the yeast-like cell forms. The flat hyphae also showed the lowest lipid-to-protein ratio (Fig. 6I) among all cell forms. It can be noticed that in the flat hyphae, absorbance intensities for the protein peak at 1650 cm^−1^ and all lipid associated peaks except 3010 cm^−1^ and 1708 cm^−1^ showed homogeneous distribution (Fig. 6B–E, G), while for the peaks at 3010 cm^−1^, 1708 cm^−1^ and 1600 cm^−1^, absorbance intensities were distributed heterogeneously, with high and low intensity areas (Fig. 6A, F, H).

The highest absorbance intensities for flat and swollen hyphae cells were observed for the peak at 1742 cm^−1^ (Figs. 6E, 7E). In the swollen hyphae, areas with high concentration of proteins at 1650 cm^−1^ were co-localized with the areas that were rich in free fatty acids (FFAs), which can be detected at 1708 cm^−1^ (Fig. 7F, G). It might be an indication of an active lipogenesis process taking place in these areas. In addition, high absorbance intensities for peaks at 2955 cm^−1^, 2925 cm^−1^ and 2850 cm^−1^ (Fig. 7B–D) showed high and evenly distributed absorbance intensities all over the swollen hyphae, while the signals at 1742 cm^−1^, 1708 cm^−1^, 1650 cm^−1^ and 1600 cm^−1^ (Fig. 7E–H) seem to be more heterogeneously distributed along the same hyphae.

The yeast-like cells of *M. circinelloides* exhibited the highest absorbance intensities for lipid-associated peaks at 3010 cm^−1^, 2955 cm^−1^, 2925 cm^−1^, and 1742 cm^−1^ in comparison to the hyphae-like cell forms (Fig. 8A–C, E). High absorbance at 3010 cm^−1^ (Fig. 8A), which is linked to =C–H stretching and represents the level of unsaturation in lipids, could be yet another indication of different lipogenesis stages occurring in different cell forms of *M. circinelloides*. At the same time, the signal for the protein peak at 1650 cm^−1^ within yeast-like cells was very low compared to other cell forms (Fig. 8G).

Lipid-to-protein ratio, which is commonly used in IR spectroscopy to estimate lipid yield and calculated in this study as the ratio of absorbance intensities at 1742 cm^−1^ and 1650 cm^−1^, was the lowest in the flat hyphae, with only few areas showing a significant increase in the ratio intensity (Fig. 6I). The swollen hyphae were mainly composed of lipids and characterized by more uniform distribution of lipid-to-protein concentration, except for a large cavity filled presumably by FFAs and proteins and lacking TAGs (Fig. 7I). The yeast-like cells seem to be comprised mostly of lipids having the largest lipid-to-protein ratio among all studied cell forms (Fig. 8I).

### Nanoscale analysis of lipid droplet areas in fungal hyphae by AFM-IR nanospectroscopy

AFM-IR nanospectroscopy allows obtaining chemical information of intact biological systems at a subcellular level [31, 32]. AFM microscopy is well known to produce high-quality topography images of samples with nanometric resolution. Recently, AFM systems were for the first time combined with IR spectroscopy enabling simultaneous acquisition of chemical and morphological information at the level of single cell organelles [31, 32]. In particular, AFM-IR measurements can provide insights into the structural and chemical differences between single lipid droplets located in different parts of a fungal cell, as well as on the lipid droplet homogeneity or heterogeneity.

**Fig. 9:**
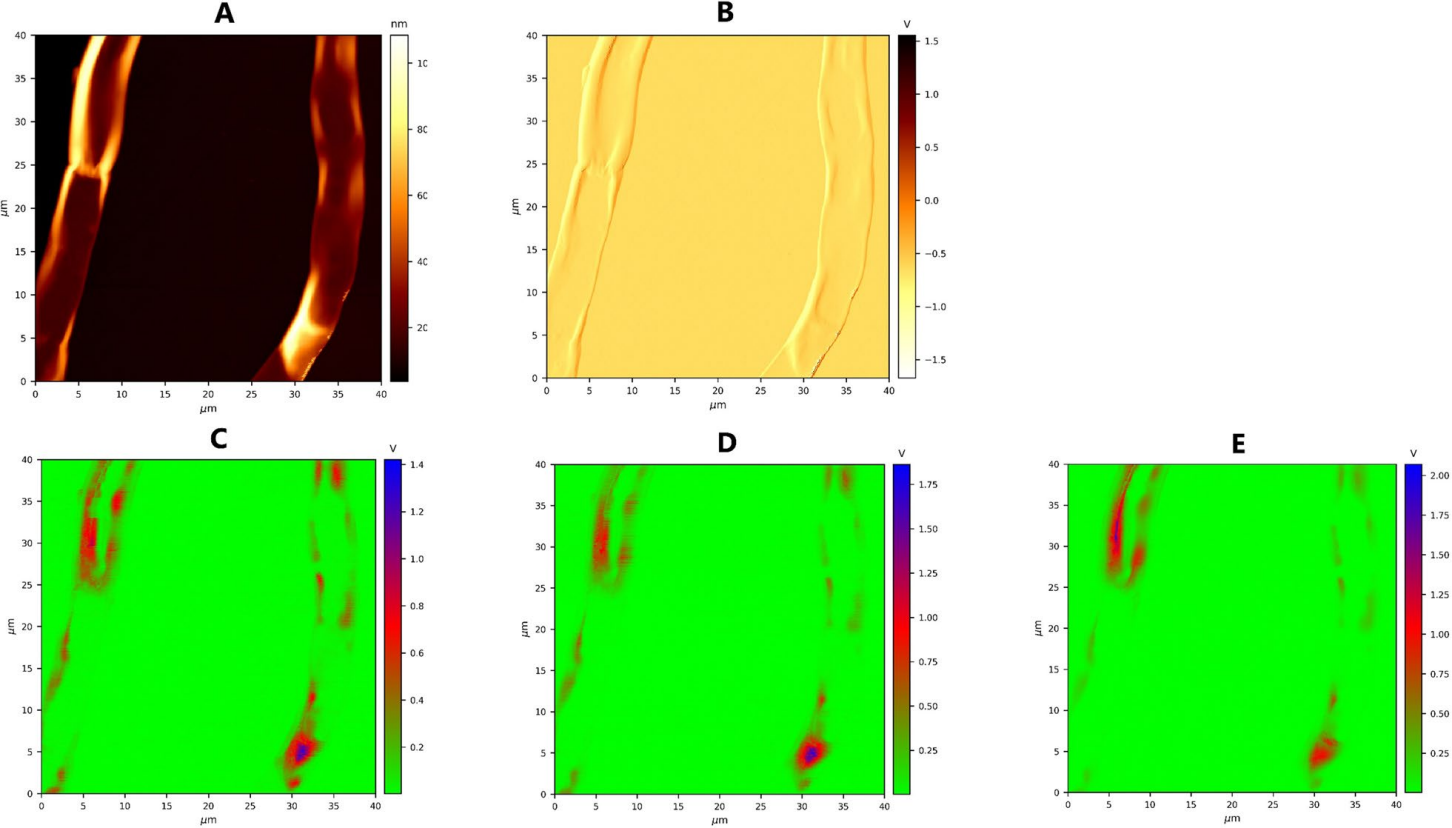
AFM-IR images of *Mucor circinelloides* fungus*,* control sample, flat hyphae cells: **A** AFM topography image at 1600 cm^−1^, **B** AFM-IR deflection image at cm^−1^, **C** AFM-IR absorption image at 1600 cm^−1^, **D** 1708 cm^−1^, **E** 1742 cm^−1^

**Fig. 10:**
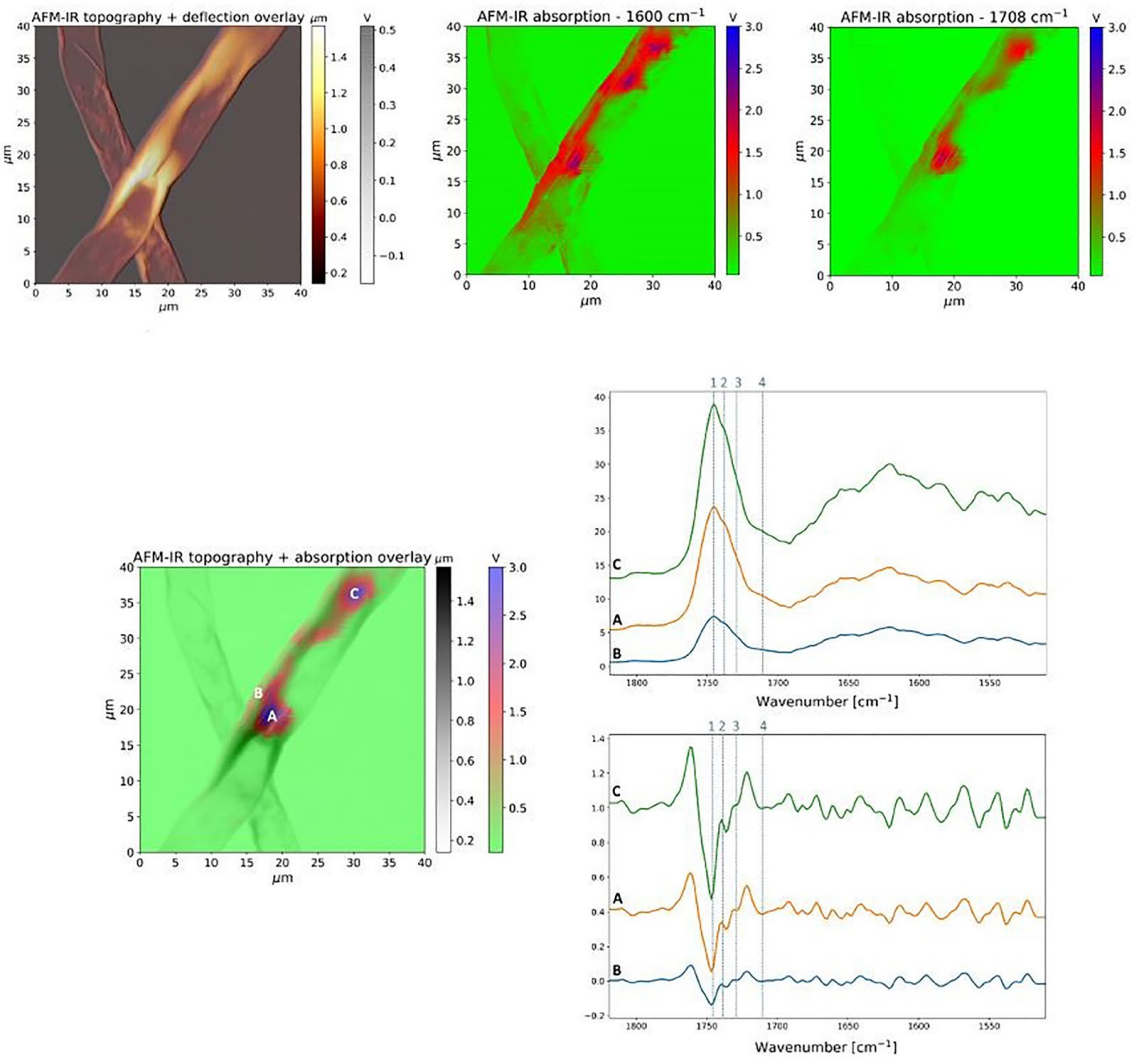
AFM-IR images of *Mucor circinelloides* fungus, control samples, swollen hyphae cells: **A** overlay of AFM topography and AFM deflection images, **B** AFM-IR absorption image at 1600 cm^−1^, **C** AFM-IR absorption image at 1708 cm^−1^, **D** overlay of AFM topography and AFM-IR absorption images at 1742 cm^−1^, **E** AFM-IR spectra, **F** AFM-IR spectra after 2nd derivative and Savitzky-Golay smoothing

In this study, AFM-IR nanospectroscopy was used for the analysis of lipid droplet areas in the flat and swollen hyphae of *M. circinelloides*. It was challenging to obtain reliable AFM-IR data from yeast-like cells as the AFM tip tends to lose contact with a probed surface that is too thick in z-direction (> 2–3 µm), i.e. out of the sample measurement plane. As a result, AFM-IR spectral maps of yeast-like cells were not recorded. Both sample Pi1 and sample Pi0, which were cultivated under presence (Pi1) or absence (Pi0) of inorganic phosphorus in growth media, were examined. As long as the high-resolution spectral maps of relatively large areas are obtained, AFM-IR measurements in imaging mode is a time-consuming process (approx. 30 min per image for the nanoIR experimental setup), therefore only a few areas within each sample could be probed. First, we obtained a number of point spectra within different areas of flat and swollen fungal hyphae in order to identify the most prominent peaks related to lipids (Figs. 9 and 10). The highest absorbance was detected at 1742 cm^−1^ and 1708 cm^−1^, at peaks that are associated with the total content of TAGs and free fatty acids, respectively (Figs. 9 and 10). A shoulder around 1735 cm^−1^ can be attributed to mono- and diglycerides (MAGs and DAGs), metabolic precursors of TAGs, which are known to exhibit absorption bands at 1730 cm^−1^ and 1710 cm^−1^ for DAGs, at 1730 cm^−1^ for MAGs, respectively. A peak around 1600 cm^−1^ was detected in both flat and swollen hyphae, which was observed as a shoulder band in HTS- and ATR-FTIR spectra at the macroscale level. It was hypothesized that due to the hyphae appearing empty on AFM topography images, their thickness (~ 200 nm for swollen hyphae in sample Pi0 and ~ 150 nm for flat hyphae in sample Pi1/CS) is determined primarily by the thickness of the cell wall, which makes it possible to detect asymmetric COO- vibrations from beta-glucans present in the fungal cell wall. As the QCL laser used for this study could only be tuned from 1810 to 1500 cm^−1^, we were not able to verify our hypothesis by examining a spectral range between 1300 and 900 cm^−1^ that is characteristic of polysaccharides. Another hypothesis was that absorbance at 1600 cm^−1^ might also give evidence to the presence of ionized free fatty acids in fungal cytoplasm [33].

For each area within the sample, AFM-IR spectral maps at 1600 cm^−1^, 1708 cm^−1^ and 1742 cm^−1^ were obtained. Comparing AFM topography images of flat and swollen hyphae, it could be seen that lipid droplet areas represented by the slight swelling are located close to the edges in the flat hyphae (Fig. 10), while in swollen hyphae areas of overall swelling could be observed in the center (Fig. 9). It lets us hypothesize that swelling of hyphae and possible formation of yeast-like cells is driven by the different stages of lipid accumulation in the fungus*.*. Absorbance intensities for the TAG-related peak at 1742 cm^−1^ were higher than FFAs-related peak at 1708 cm^−1^ within lipid droplet areas in both flat and swollen hyphae (Figs. 9C, D and 10C, D). Furthermore, the highest absorbance for all three measured peaks was observed in the center of lipid droplet areas. Some level of possible heterogeneity could be seen for lipid droplet areas in flat hyphae, where two lipid droplet areas showed difference in absorbance intensity for peaks at 1742 cm^−1^ and 1708 cm^−1^ (Fig. 10C, D).

## Discussion

FTIR spectroscopy measurements of bulk biomass are currently the most common approach in vibrational spectroscopy to estimate the relative amount of lipids, proteins, and other constituents in intact microbial biomass [3, 16, 20]. In the first part of this paper, we examined the most commonly used FTIR methods for the analysis of bulk fungal biomass. By comparing spectra of *Mucor circinelloides* obtained with two macroscale FTIR measurement techniques, HTS-FTIR and ATR-FTIR, we observed significant differences mainly in two parameters: (1) relative peak intensity and (2) peak shifts. The difference in relative peak intensity is due to the physics of the sampling mode employed in a particular FTIR measurement technique. In transmission-based HTS-FTIR measurements, an optical path length of an IR beam through a sample is determined by the sample thickness, and it is constant within the measured point, while in ATR-FTIR measurements, the actual penetration depth is a function of the wavenumber and therefore becomes larger with smaller wavenumber (frequency) values [35]. Penetration depth of IR radiation for biological materials such as fungi has been estimated to vary between ~ 1.1 µm at 1742 cm^−1^ to ~ 1.8 µm at 1100 cm^−1^ [36]. In ATR-FTIR measurements of dried intact fungal biomass, the penetration depth of IR radiation is large enough to probe the fungal cell membrane and cell wall, which is typically several micrometers thick. However, the intensity of the evanescent field is not strong enough to provide a comparable absorption signal for intracellular components [37]. Fungal hyphae that have accumulated larger amounts of lipids due to specific cultivation conditions triggering lipid accumulation have a thicker cell wall compared to hyphae grown under less favorable conditions [6, 24]. While cell wall and cell membrane are mainly responsible for the absorption features in ATR-FTIR spectra of dried intact fungal biomass, intracellular components such as lipids, proteins, etc. may still contribute to a minor degree depending on the thickness of the cell wall. Due to the ATR-FTIR measurement principle, signal saturation cannot occur, while in HTS-FTIR transmission measurements, samples with high concentration in a cell suspension transferred to HTS-FTIR silicon plates can easily result in total absorption of radiation and saturation features in spectra. Therefore, ATR-FTIR measurements can be performed with a simple and fast sample preparation protocol without optimizing the film thickness as required for HTS-FTIR measurements.

Peak shifts in ATR-FTIR spectra as compared to HTS-FTIR spectra can be explained by a dispersion of the refractive index: rays of shorter wavelengths (i.e. higher wavenumbers) are refracted less than those of longer wavelengths (i.e. lower wavenumbers), which means that the refractive index is higher when approaching the shorter end of the mid-IR [38]. Since the penetration depth depends on the refractive index [35], a peak shift is observed in ATR-FTIR spectra. It should be noted that biological samples in general—and fungi in particular—are highly heterogeneous, and different regions of fungal hyphae might have different refractive index values. As a result, variation in the penetration depth due to differences in the refractive index are expected [36]. Different spectral resolution used in HTS- and ATR-FTIR measurements (± 6) cm^−1^ and 4 (± 4) cm^−1^, respectively might also contribute to the precision a peak position could be determined with, and to the ability to resolve two closely located peaks.

In spite of the different physical phenomena occurring in transmission- and reflectance-based FTIR experiments, HTS-FTIR and ATR-FTIR spectra of *M. circinelloides* show high compatibility. The use of a particular FTIR technique should be defined by the purpose of the screening study. For instance, HTS-FTIR spectroscopy is better suited for the estimation of lipid yield and fatty acid profiles in oleaginous fungi. ATR-FTIR spectroscopy, in turn, is more appropriate for monitoring sample dynamics over time, as well as for examining composition of fungal cell wall and cell membrane. HTS-FTIR experiments, as the name suggests, permit large-scale screening to estimate best lipid producers among a high number of fungal strains and cultivation conditions, and optimize lipid production processes [1, 6, 15, 23, 24, 39, 40]. ATR-FTIR measurements are performed for one sample at a time and require washing of the ATR surface after each measurement.

Macro-FTIR techniques presented above are therefore complementary with respect to the cell components they address and to the sample form to be analyzed. When combined with wet chemical techniques, both HTS-FTIR and ATR-FTIR measurements can be calibrated and serve as quantitative methods for lipid analysis. While chromatography-based analysis allows the direct quantification of lipids, macroscale vibrational spectroscopy analysis provides a fingerprint of the overall chemical composition of the fungal biomass without any need for extraction and tedious sample preparation, which is well suited for high-throughput screening.

While FTIR-based macroscale screening is highly suitable for the optimization of microbial lipid production, it is desirable to be able to explore physiology of dimorphic fungi on a cellular level. It has been shown that lipogenesis in different cell forms of dimorphic fungi may differ considerably [41]. Since the cell form of dimorphic fungi is highly correlated with cell wall chemistry, the co-production of valuable cell wall polymers and lipids strongly depends on the morphology of cells [41]. Therefore, a detailed investigation of fungal lipogenesis at a cellular level is required for different cell forms.

Microscale analysis by FTIR microspectroscopy can provide detailed information about lipid accumulation in single cells, different stages of lipogenesis and biochemical differences for different cell forms of dimorphic microorganisms. In this study, microscale analysis by FTIR microspectroscopy showed significant chemical differences in lipid profiles of different cell forms (flat and swollen hyphae, and yeast-like cells) of the fungus, which indicates different lipogenesis stages occurring in different cell forms. FPA-FTIR spectral maps of flat hyphae revealed high absorbance intensities for both protein-associated peak at 1650 cm^−1^ and lipid-associated peaks, while lipid-to-protein ratio was the lowest in flat hyphae. Based on these observations, we hypothesize that flat hyphae are characterized by the initial stages of lipogenesis, when synthesis of different free fatty acids takes place, TAGs synthesis starts, and a wide range of enzymes is involved. TAGs-related peak exhibited homogeneously distributed intensity, while FFAs peak intensities were distributed heterogeneously, confirming reports that lipogenesis-active sites in the cell are very often associated with the endoplasmic reticulum, which has heterogeneous morphology.

In swollen hyphae, a significant increase in absorbance values for lipid-related peaks and a decrease for the protein peak could be observed, indicating an active synthesis of TAGs and FFAs. According to FPA-FTIR spectral maps, yeast-like cells were characterized by the highest lipid-to-protein ratios and higher absorbance values at 3010 cm^−1^ indicating a higher total lipid content and higher content of unsaturated fatty acids compared to other cell forms within the same cell culture. It can be concluded that yeast-like cell forms are the most desirable for high lipid yield production, and cultivation conditions should be optimized with respect to maximizing the amount of yeast-like cells in the resulting fungal biomass.

In order to produce fungal biomass with a targeted lipid composition, there is a need for a better understanding of the homogeneity and/or heterogeneity between and within the lipid droplets, its consistency and flexibility [40]. One of the main reasons for the current knowledge gap is the absence of analytical techniques to perform nanoscale lipid analysis at a single lipid droplet level.

AFM-IR nanospectroscopy is a brand new technique that offers a unique opportunity for the nanoscale analysis at the level of single lipid droplets. It can provide specific information on the formation of lipid droplets, as well as on their morphology and chemical homogeneity/heterogeneity. To the authors' knowledge, this study is the first application of AFM-IR nanospectroscopy on studying single lipid droplets in oleaginous fungi. According to AFM-IR spectra taken at different points within lipid droplets, the highest absorbance signals were observed from two lipid-related peaks at 1742 cm^−1^ and 1708 cm^−1^ that are characteristic of TAGs and FFAs content, respectively. A shoulder at 1735 cm^−1^ that corresponds to MAGs and DAGs might give evidence to metabolic activity taking place within lipid droplet areas in fungal cells, where MAGs and DAGs are synthesized from FFAs or from TAGs remobilization (broken down to form DAGs, MAGs and FFAs). In addition, a peak at 1600 cm^−1^ corresponding to asymmetric COO– stretching, which was clearly identifiable only in AFM-IR spectra, might be related to either ionized free fatty acids or beta-glucans present in the fungal cell wall. AFM-IR spectral maps allowed observing edge-oriented localization of lipid droplet areas with the highest absorbance intensities for lipid-associated peaks at their center, which confirms similar information obtained from FPA-FTIR spectral maps of flat hyphae-like cells. Furthermore, AFM-IR analysis of two lipid droplets within the same fungal hyphae showed that lipid profiles of their cores differ. Therefore, it comes as the first evidence of heterogeneity within and between lipid droplets in oleaginous fungi. Two possible explanations of lipid droplet heterogeneity might be given: (i) It might be an indication of the TAGs or FFAs synthesis happening in the core of lipid droplets, while active transport of building blocks needed for the synthesis takes place in their outer parts; (ii) The concentration of the analyte (TAGs, FFAs) is the largest in the center of the examined area. It is important to understand how to regulate the ratio of TAGs and FFAs within lipid droplets by influencing lipogenesis stages and thus increasing TAGs or FFAs yield. AFM-IR analysis of lipid droplets in yeast-like cells deposited on the CaF_2_ slides was not successful due to the extraordinary high lipid content in this cell forms leading to the high sample thickness and, therefore, different sample preparation approaches need to be applied.

## Conclusions

Vibrational spectroscopy techniques provide a comprehensive information on lipogenesis in the different cell forms of dimorphic and toleaginous fungi. In this study we demonstrated that, both, transmission and reflectance measurement modes of the bulk fungal biomass can be used to evaluate the total lipid content. Single cell spectroscopic measurements showed distinct variation in lipogenesis between different cell forms of dimorphic oleaginous *Mucor circinelloides*, where amount of lipids increases in the following order: flat hyphae—swollen hyphae—yeast-like cells, and yeast-like cells can be considered as the fatty ones. Single lipid droplet measurements by nanospectroscopy gave a clear indication of droplet-to-droplet and within-droplet heterogeneity which needs to be studied further.

## Methods

### Cultivation of oleaginous *Mucor circinelloides* and preparation for spectroscopic measurements

*Mucor circinelloides* VI 04473 was provided by the Faculty of Veterinary Medicine, Norwegian University of Life Sciences (Ås, Norway). The cultivation of the *M. circinelloides* VI 04,473 was performed in two steps as described in Dzurendová et al. [22] and Kosa et al. [24]: (1) cultivation on agar plates for a spore inoculum preparation, and (2) cultivation triggering lipid accumulation by using nitrogen-limited broth media and different amounts of inorganic phosphorus salts (Pi). For the preparation of the spore inoculum, malt extract agar (MEA) was used. MEA was prepared as described in S. Dzurendová et al. [22]. Agar cultivation was performed for 7 days at 25 °C. Fungal spores were harvested with a bacteriological loop after the addition of 10 mL of sterile 0.9% NaCl solution. The main components of the nitrogen-limited broth media were prepared according to the previously published studies on the screening of *Mucoromycota* fungi Dzurendová et al. [22] and Kosa et al. [24]. The concentration of inorganic phosphate salts used in the cultivation media are listed in Table 1. Sample Pi1, which was cultivated under the most optimal conditions for lipid accumulation in *Mucor circinelloides* according to Kosa et al. [22] and Dzurendová et al. [39], was selected as a control sample (CS).

Cultivation in the nitrogen-limited broth media was performed in the Duetz-MTPS (Enzyscreen, Heemstede, Netherlands), consisting of 24-square polypropylene deep well microtiter plates, low evaporation sandwich covers and extra high cover clamp system, which were mounted into the shaking incubator MAXQ 4000 (Thermo Scientific, Oslo, Norway). 7 ml of the sterile broth media was transferred into the autoclaved microtiter plates and each well was inoculated with 50 µL of the spore suspension. Cultivations were performed for 7 days at 25 °C and 400 rpm agitation speed (1.9 cm circular orbit).

For HTS-FTIR spectroscopy measurements, washed fungal biomass was homogenized by bead beating using tissue homogenizer (Percellys Evolution, Bertin instruments, France), and 8 µl of homogeneous fungal cell suspension was deposited on 384-well silicon plate and dried at room temperature. Three technical replicates were prepared for each sample. For ATR-FTIR spectroscopy measurements, washed fungal biomass was placed on Petri dishes and dried at room temperature for 24 h. Three technical replicates were prepared for each sample. For FPA-FTIR microspectroscopy measurements, washed fungal biomass was deposited on CaF_2_ slides and accurately distributed to isolate single hyphae. The sample was then dried at room temperature. For AFM-IR nanospectroscopy measurements, dried fungal biomass was rehydrated by adding 8 ml of distilled water and 20 ml of the suspended diluted solution was deposited on the CaF_2_ slides and dried at room temperature.

### Vibrational spectroscopy measurements

#### HTS-FTIR and ATR-FTIR spectroscopy measurements

HTS-FTIR and ATR-FTIR spectroscopic measurements of the fungal biomass were performed by a Vertex 70 spectrometer (Bruker, Massachusetts, USA). For HTS-FTIR measurements, each sample was measured in a range from 4000 to 500 cm^−1^, with a spectral resolution of 6 cm^−1^ and an aperture of 5 mm. A resulting spectrum was obtained as an average of 64 scans. A background spectrum was recorded as a spectrum of an empty microtiter plate prior to probing of every sample plate. For ATR-FTIR measurements, a diamond prism was used as a substrate in a single-reflection mode. Each sample was measured in a range from 4000 to 400 cm^−1^ with a spectral resolution of 4 cm^−1^ and 32 scans to produce a resulting spectrum. Before every sample measurement, a background spectrum of an empty ATR crystal was acquired as an average of 32 scans. Each biological replicate was measured in three technical replicates and averaged afterwards. For six conditions related to different phosphorus amounts, HTS-FTIR spectral measurements were carried out during the same day, while ATR-FTIR measurements were performed during 7 days to assess chemical stability of the fungal biomass. For both, HTS-FTIR and ATR-FTIR measurements, a resulting spectrum was calculated as logarithm of ratio between the sample spectrum and the background spectrum, taken with a negative sign. In total, 18 HTS-FTIR spectra and 126 ATR-FTIR spectra were obtained for *M. circinelloides* fungus.

#### FPA-FTIR microspectroscopy measurements

FPA-FTIR measurements were performed at the Synchrotron SOLEIL facility (Saint-Aubin, France). A high magnification FTIR-FPA was used, 128 scans per point, with the resulting image size of 84 per 84 pixels, 0.7 m per pixel. All measurements were performed in a transmission mode. The reflection mode was used to find measurement regions and the background region. Background measurements were performed once per sample. Three areas of interest were assessed using FTIR microspectroscopy, and 16,384 spectra were obtained for each area, resulting in total of 49,152 spectra.

#### AFM-IR nanospectroscopy measurements

For AFM-IR measurements, nanoIR commercial experimental setup (Anasys Instruments, Santa Barbara, CA, USA) was used. A quantum-cascade laser (QCL) (MIRcat-1100, Daylight Solutions, San Diego, CA, USA) tunable between 1822 and 1510 cm^−1^ was used as an IR source. Laser power was tuned between 10 and 50% of its maximum depending on the region of interest to reduce the sample damage caused by the heating. CaF_2_ slides were placed onto a CaF_2_ prism and irradiated using a bottom-up illumination scheme as implemented in nanoIR. Thermal expansion caused by the laser radiation was detected by a Cr-Au AFM cantilever tip (HQ:CSC38, MikroMasch, Bulgaria) with a spring constant k = 0.3–0.9 N/m, and initial force F = 10–20 nN. AFM-IR measurements were performed in a resonance-enhanced mode [43, 44]. Laser repetition rate was chosen to match one of the contact resonance frequencies of the AFM cantilever (usually—2nd or 3rd resonance frequency of the AFM cantilever) using a phase-locked loop (PLL) mode with parameters for integral and proportional gains adjusted to 6 and 10, respectively. Gain parameters were set separately for AFM topography and IR absorption measurements.

AFM-IR spectra were obtained for different points within the areas of interest. The laser was tuned from 1800 to 1510 cm^−1^ with a 1 cm^−1^ step, and 256 laser pulses were generated for the resonance to stable. Afterwards, spectra were collected at room temperature. A so-called background spectrum was acquired for each wavenumber within the studied wavenumber range at 100% laser power to be later used for the normalization of AFM-IR spectra and spectral maps. AFM-IR spectral maps were collected along with AFM topography and deflection images for three fixed wavenumbers of interest: 1600 cm^−1^, 1708 cm^−1^ and 1742 cm^−1^ corresponding to free fatty acids in ionized forms/beta-glucans, free fatty acids (FFAs) and triacylglycerides (TAGs), respectively. All measurements were conducted in a contact mode, with 256 lasers pulses produced to be averaged as a resulting data pixel value. In total, 30 spectral maps (3 maps per each area, 5 areas per each sample, 2 samples), along with the same number of corresponding AFM topography and deflection maps were collected. It took around 30 min to measure one batch of AFM-IR image data (topography, deflection, absorption) per area.

### Data analysis

All spectroscopic data were analyzed using in-house developed algorithms written in Python. For the assessment of influence of different drying times on ATR-FTIR spectra of *M. circinelloides*, ATR-FTIR spectra were pre-processed in the following way: (1) Second derivative was calculated using a Savitzky-Golay (SG) filter [45] (window size = 15, order of a polynomial to be fitted = 2); (2) Extended multiplicative signal correction (EMSC) [47] was used to remove baseline shift and scaling effects, as well as first- and second-order polynomial dependency. Aforementioned pre-processing steps were applied to ATR-FTIR spectral data within the following spectral regions: from 4000 to 700 cm^−1^ (whole spectra), from 3050 to 2800 cm^−1^ and 1800 cm^−1^ to 1700 cm^−1^ (lipid region), 1700 cm^−1^ to 1200 cm^−1^ (combined protein and mixed region), and 1200 cm^−1^ to 700 cm^−1^ (polysaccharide and polyphosphate region).

To estimate the influence of storage time on the biochemical profile of *M. circinelloides* samples, ANOVA–PCA of ATR-FTIR spectra was employed. ANOVA-PCA partitions variation by separating different effects in a non-correlating way [47]. In case of the ATR-FTIR experiment, estimating the influence of storage time was made possible by separating it from the influence of different cultivation conditions. Two design matrixes were created to describe the experiment: one with sample grouping according to the storage time points, another with sample grouping according to the different concentrations of phosphorus in the growth media. Each design matrix has a shape of (*n_spectra*, *n_factor_levels*), where (*n_spectra*) is a total number of ATR-FTIR spectra, and (*n_effect_levels*) is a number of distinct values (*levels*) corresponding to each effect. Design matrixes were used to calculate estimates for both effects and their interactions. Resulting partitions were subjected to PCA together with the residual matrix comprising the information not assigned to any effect.

For the comparison of HTS- and ATR-FTIR spectra, ATR-FTIR spectra of *M. circinelloides* corresponding to the first day of storage were used. The following pre-processing steps were employed: (1) An intersection of HTS- and ATR-FTIR wavenumbers was obtained by aligning, both, HTS- and ATR-FTIR datasets to perform peak-to-peak comparison; (2) EMSC correction (linear and quadratic effects) was applied to both datasets separately; (3) The most prominent peaks were selected using *find_peaks* method from the Signal Processing module of SciPy, Python’s open-source library for scientific computing [49]. Peak selection and their subsequent comparison has been performed within several wavenumber regions, such as: (1) combined lipid region: from 3050 to 2800 cm^−1^ (lipids) and from 1800 to 1700 cm^−1^, (2) protein region: from 1700 to 1500 cm^−1^, (3) mixed region: from 1500 to 1200 cm^−1^, and (4) polysaccharide combined with polyphosphate region: from 1200 to 700 cm^−1^. A Savitzky-Golay filter (window size = 15, polynomial order = 2, derivative order = 2) was applied to the mean HTS- and ATR-FTIR spectra corresponding to each sample, and resulting spectra were used for the peak selection.

Differences between exact positions of the same peaks in HTS- and ATR-FTIR spectra were calculated. Absorbance ratios between the closest counterparts of the following peaks were calculated in both HTS- and ATR-FTIR mean sample spectra: 1742 cm^−1^ and 1650 cm^−1^, 3010 cm^−1^ and 1742 cm^−1^, 3010 cm^−1^ and 1650 cm^−1^.

For the analysis of FPA-FTIR spectral data the following pre-processing pipeline was established:

1. Removal of noisy and non-informative wavenumber regions
  For each dataset, certain wavenumbers were excluded to avoid noisy and non-informative absorbance signals present at certain wavenumbers from having a detrimental effect on the subsequent data analysis. As such, there is a strong water-absorbing region with no lipid-related signatures above 3200 cm^−1^, and absorbance values below 1000 cm^−1^ contained negative values due to the acquisition errors. Therefore, a typical working range for every FPA-FTIR hyperspectral image was selected between 3200 and 1000 cm^−1^.
2. Removal of outliers
  Spectral data were examined for outliers in each dataset that corresponded to noisy spectra and dead pixels. For each spectral map, a data pixel was classified as an outlier if its value was more than three scaled median absolute deviations (MAD) away from the median absorbance value of the spectral map. Furthermore, pixels that maintained anomalously large values all over the hyperspectral dataset were also detected as outliers. Values of outlier pixels were replaced by the mean values of the neighboring non-outlier pixels for each spectral map separately.
3. FPA-FTIR mage segmentation
  FPA-FTIR spectral images that correspond to the most prominent lipid- and protein-related peaks such as 3010 cm^−1^, 2955 cm^−1^, 2925 cm^−1^, 2855 cm^−1^, 1742 cm^−1^, 1708 cm^−1^, and 1650 cm^−1^, were selected for subsequent image segmentation to separate hyphae pixels from non-informative slide background pixels. To do that, selected FPA-FTIR spectral data were scaled (StandardScaler from *sklearn preprocessing*) module with default parameters was employed) and then subjected to PCA analysis. Principal components corresponding to 99% of total variance were processed with *k-means* clustering, which utilized *k-means*++ initialization scheme to detect two clusters.
4. DSAE correction of FPA-FTIR spectral data and image comparison
  A descattering autoencoder [49], which was pre-trained on a set of infrared spectra of *M. circinelloides* that were scatter-corrected by the Mie Extinction Extended Multiplicative Signal Correction (ME-EMSC) algorithm, was used to obtain scatter-free FPA-FTIR spectral data. Only spectra that correspond to fungal hyphae were processed by the DSAE to yield smoothed and scatter-free spectra. Resulting data were reshaped as images and plotted alongside each other. A scale was adjusted for each image separately to make the chemical differences within a spectral map more pronounced. Furthermore, a ratio map was calculated for each area of interest using spectral maps at 1742 cm^−1^ and 1650 cm^−1^.
5. Analysis of AFM-IR spectra and spectral maps
  The following pre-processing pipeline was employed for AFM-IR spectra: (i) Spectra corresponding to the same acquisition point were averaged (typically 4 to 5 spectra were collected per point); (ii) Spectra were smoothed using Savitzky-Golay filter with the window size of 15 and a polynomial order of 1; (iii) EMSC correction was used to remove physical effects [50–53].
6. AFM-IR spectral maps
  For every area of interest, AFM-IR spectral images acquired at 1600 cm^−1^, 1708 cm^−1^ and 1742 cm^−1^, along with the corresponding AFM topography and deflection images, were pre-processed and analyzed as follows:
  - Removal of artifacts: AFM topography images were corrected to remove background tilt (slope) and height shifts. The slope, which might be induced by minor surface irregularities or scanner bow generated by the motion of the free end of the AFM cantilever following an ark, was corrected by fitting a polynomial to the topography data. Usually, a simple planar fit is employed, but if a curvature is present, higher order polynomials can be used. Height shifts that are due to different Z-position values corresponding to different scan lines were removed by subtracting a median height value for each line. Furthermore, AFM-IR absorption artifacts due to the temporary loss of contact between the sample and the AFM tip—and therefore loss of the resonance frequency needed for a strong absorption signal to be produced—were replaced with the average value of the neighboring pixels.
  - Unit change: Topography data was converted to micrometers to avoid extremely high values while calculating absorption vs. topography ratio images later.
  - Compensation of thermal drift: A drift typically occurs between several AFM images acquired at different time points because of the temperature fluctuations and tip-induced artifacts. It must be noted that since AFM topography and AFM-IR absorption measurements are performed simultaneously, both of the resulting data types are affected by the drift. To compensate for the drift, an image registration algorithm was employed. For each area of interest, an AFM topography image at 1600 cm^−1^ served as a fixed image, and the other topography images—as moving ones. A rigid transformation that accounts for translation and rotation of the moving image relative to the fixed one was estimated using the *register* function of the *StackReg* Python package for intensity-based image registration. After that, moving topography images, along with the corresponding deflection and absorption images, were transformed according to the resulting geometric transformation using the *transform* function from the same package.
  - Scaling: Negative topography values might occur due to the Z-position of the scanner not been adjusted properly during the scanning. To compensate for it, topography values were shifted towards zero so that all values became positive. Values below a certain threshold (here—5th percentile) were replaced by the threshold value itself.
  - Normalization: Absorption images were normalized to incident laser power to make them directly comparable as the QCL laser power is different for different wavenumbers in the operating range. Normalization was performed by dividing each absorption image by the laser power at a corresponding wavenumber (a so-called background spectrum).
  - Image segmentation: To separate fungal hyphae from the slide background, a multivariate *k-means* clustering was used based on the AFM topography and deflection, as well as AFM-IR absorption images for the given area of interest. Values assigned to the background were afterwards excluded from ratio calculation.
  - Absorption/topography ratio: A ratio between AFM-IR absorption images and AFM topography images taken at the same wavenumber was calculated. Logarithm of the ratio to base 10 was calculated to make differences between regions of the image more pronounced.
  - Ratio of absorption maps: A ratio between different AFM-IR absorption images (here—1742 cm^−1^ to 1600 cm^−1^ and 1708 cm^−1^ to 1600 cm^−1^) was obtained to find areas with different concentrations of TAGs and FFAs relative to proteins. Logarithm of the ratio to base 10 was calculated to amplify distinct regions within the ratio map of interest.

## Data Availability

The datasets used and/or analyzed during the current study are available from the corresponding author on reasonable request.
